# Epigenetic Modifications in Pediatric Acute Lymphoblastic Leukemia

**DOI:** 10.3389/fped.2014.00042

**Published:** 2014-05-14

**Authors:** Michael J. Burke, Teena Bhatla

**Affiliations:** ^1^Division of Pediatric Hematology-Oncology, Medical College of Wisconsin, Milwaukee, WI, USA; ^2^Division of Pediatric Hematology-Oncology, New York University Langone Medical Center, New York, NY, USA

**Keywords:** epigenetics, methylation, histone, pediatric, leukemia, ALL

## Abstract

Aberrant epigenetic modifications are well-recognized drivers for oncogenesis. Pediatric acute lymphoblastic leukemia (ALL) is no exception and serves as a model toward the significant impact these heritable alterations can have in leukemogenesis. In this brief review, we will focus on the main aspects of epigenetics, which control leukemogenesis in pediatric ALL, mainly DNA methylation, histone modification, and microRNA alterations. As we continue to gain better understanding of the driving mechanisms for pediatric ALL at both diagnosis and relapse, therapeutic interventions directed toward these pathways and mechanisms can be harnessed and introduced into clinical trials for pediatric ALL.

## Introduction

Epigenetics is the study of biochemical modifications of chromatin (1) and have been implicated in the pathogenesis of cancer (2). Epigenetic modifications to DNA are not secondary to changes to the nucleotide sequence itself but rather heritable changes affecting the activity of genes and their cellular expression. Examples include DNA methylation, histone modification, and alterations in non-coding microRNAs (miRNAs). Each of these mechanisms can alter how genes are expressed or silenced without modifying the DNA sequence. If these epigenetic modifications lead to silencing of tumor suppressor genes or activation of oncogenes then it is easy to conceptualize how leukemogenesis can occur.

Unlike chromosomal translocations or gene mutations, which are permanent, hypermethylation of gene promoters is a reversible event that could be targeted with therapeutic agents designed to alter aberrant epigenetic events. Incorporating epigenetic modifying agents into the treatment of pediatric ALL is an exciting approach that theoretically could have a significant impact in the treatment of this disease. This would be particularly true for relapse ALL, which is highly hypermethylated (3–5), and accounts for more deaths than any other pediatric disease and remains the fifth most common pediatric cancer overall (6).

In this brief review, we will focus on the three main areas of epigenetics, which have been implicated in the leukemogenesis of pediatric ALL; DNA hypermethylation, histone modification, and microRNA alterations. As we continue to gain better understanding of the driving mechanisms for pediatric ALL at both diagnosis and relapse, therapeutic interventions directed toward these pathways and mechanisms can be harnessed and introduced into clinical trials.

## DNA Hypermethylation

Gains of DNA methylation tend to occur in the gene promoter region and are one of the most studied epigenetic abnormalities in oncogenesis (7, 8). The methylation occurs at cytosine (C) bases located 5′ to guanosine (G) in a CpG dinucleotide and often in regions rich in repetitive CpGs known as CpG islands. The methyl groups are transferred to the CpG dinucleotide via DNA methyltransferases (Dnmt1, Dnmt3a, and Dnmt3b) and serve to transcriptionally silence genes downstream of the methylated promoter. When aberrant methylation occurs in a cancer cell, it typically results in hypermethylation of tumor suppressor genes. This can lead to disruption of key molecular pathways such as apoptosis, DNA repair pathways, cell cycle checkpoints, and cell differentiation as well as result in activation of metastasis/invasion pathways, drug resistance, and proliferation signal transduction (9).

Various groups have used DNA methylation studies to investigate the underlying epigenetic mechanisms in childhood leukemia. In a large cohort of 137 B-lineage and 30 T-lineage pediatric ALL cases, distinct DNA methylation signatures with significant concordant correlation of gene expression were found to be characteristic of various cytogenetic sub-types (10). In fact, a core set of epigenetically deregulated genes, common to all cases, was identified; suggesting their central role in leukemia initiation and maintenance. Likewise, DNA methylation interrogation of 69 pediatric B-ALL and 42 non-leukemic control samples revealed 325 genes hypermethylated and down regulated, and 45 genes hypomethylated and up-regulated across all the samples, irrespective of subtype (11). Furthermore, gene ontology analysis of these epigenetically deregulated genes highlighted the role of genes involved in cell signaling, cellular development, cell survival, and apoptosis. Another study investigating 764 cases of newly diagnosed ALL and 27 cases of relapse, identified 9406 predominantly hypermethylated CpG sites, independent of cytogenetic background, with each cytogenetic subtype displaying a unique set of hyper- and hypomethylated sites (12). These differentially hypermethylated CpG sites were enriched for genes in the transcriptional regulatory network such as *NANOG*, *OCT4*, *SOX2*, and *REST*. These genes are known to be regulated by a polycomb group of proteins and have been identified as targets for hypermethylation in solid tumors (13), leukemia (14), and lymphoma (15).

MLL-rearranged infant leukemia is one specific ALL subtype that has been shown to exhibit distinct promoter hypermethylation (16–19). Stumpel and colleagues identified a distinct DNA methylation pattern dependent on the presence and type of MLL-fusion partner in a cohort of 57 newly diagnosed infant ALL patients (19). In addition, the degree of hypermethylation appeared to correlate with a higher risk of relapse among infants carrying *t*(4;11) or *t*(11;19) translocations. In another study of 5 MLL-rearranged infant ALL samples, genes known to be involved in oncogenesis and tumor progression (*DAPK1*, *CCR6*, *HRK*, *LIFR*, and *FHIT*) were differentially methylated suggesting a role in the leukemogenesis of MLL-rearranged ALL (17). As well, four of five genes that were hypermethylated and silenced were able to be re-expressed *in vitro* when exposed to DNMTi and regain their functional roles, thus pointing to the clinical potential epigenetic therapy may have in the treatment of infant leukemia.

Relapsed ALL is a highly aggressive disease marked predominantly by drug resistance (20). Efforts are currently being undertaken to identify the role of epigenetic mechanisms in driving relapse and chemoresistance (3). Genome-wide DNA methylation profiling performed on 33 matched relapse-diagnosis pairs demonstrated that the relapsed genome was distinctly more hypermethylated compared to matched samples at diagnosis (3). In this study, 1147 CpG sites corresponding to 905 genes were differentially hypermethylated at relapse. About a third of these genes exhibited concordant down-regulation of mRNA expression. Many of the known regulators of the Wnt pathway were hypermethylated and down regulated at relapse, including inhibitors of the β-catenin/TCF/LEF activity, as well as *APC*, *WT1*, cadherins (*CDH1*, *CDH11*), and SOX genes (*SOX2*, *SOX8*, *SOX11*, *SOX21*). Interestingly, *PTPRO*, a negative feedback inhibitor of the Wnt pathway that binds to Wnt and blocks its association with other receptors (21), was also hypermethylated and down regulated. This suggests that the Wnt pathway is over-activated at relapse and that aberrant DNA methylation may play a significant role in the activation of this pathway in relapsed ALL (3). Re-expression of these hypermethylated and down regulated genes was observed when leukemia cell lines were treated with decitabine. As well, enhanced chemosensitivity was observed when ALL cell lines and primary patient ALL samples were pretreated with decitabine followed by conventional cytotoxic chemotherapy (4).

In summary, DNA hypermethylation appears to play a significant role in the leukemogenesis of ALL and may be an important contributor toward relapse. As more studies interrogate the specific genes and or pathways influenced by hypermethylation in pediatric ALL, we will gain further insight toward strategies to therapeutically target these aberrant epigenetic changes and hopefully begin to make a greater impact in the treatment of this disease.

## Histone Modifications

Histones are small basic proteins involved in the spatial organization of DNA within the nucleus. The chromatin environment influences the “on–off” transcriptional states of a gene depending on the post-translational modifications of the histone proteins (22). Numerous covalent histone tail modifications, the most prominent being methylation and acetylation, can directly affect gene transcription (23). These modifications are highly specific for the particular amino acid position on the N-terminal tails of the histones. For example, H3K4me3, H3K9 acetylation, H3K14 acetylation, and H3K79me2 are associated with open chromatin structures and linked with transcriptional activation, while H3K9me3 and H3K27me3 are associated with closed chromatin, and hence transcriptional repression. These histone marks are regulated by the balance between competing enzymes such as the histone lysine methyltransferases (HKMTs) and histone demethylases (HKDMs), and the histone acetyltransferases (HATs) and histone deacetylases (HDACs) (24). Moreover, multiple histone modifications can be associated with critical regulatory elements of transcription such as enhancers, which can determine cell fate and differentiation (23, 25).

Mutations in epigenetic modifying genes are common in hematologic malignancies, including ALL (26–31). These mutations can result in a gain or loss of function of key genes known to regulate histone marks. Jaffe and colleagues, in pediatric ALL cell lines, have used global chromatin profiling, a tandem mass spectrometry strategy, to measure levels of histone modifications on bulk chromatin (29). In this work, a novel cluster of cell lines with a specific epigenetic signature was identified, characterized by increased dimethylation of histone H3 at lysine 36 (H3K36me2) and decreased unmodified H3K36. Approximately half of the cell lines in this cluster harbored the *t*(4;14) translocation, which is known to induce overexpression of NSD2 (24, 32, 33). NSD2 is a member of the HKMTs that catalyze the conversion of unmodified H3K36 to mono- and dimethylated forms (28). Upon targeted sequencing in an extensive patient sample set, NSD2 mutations were found to be enriched in *ETV6-RUNX1* and *TCF3-PBX1* sub-types of pediatric B-ALL, while no mutations were identified in 30 adult ALL samples. These were gain-of-function mutations and their overexpression led to a global increase in H3K36me2, with concomitant decrease in H3K27me3. Similar results were reported by others (34), showing these mutations affect expression of a number of genes involved in normal lymphoid development.

Accumulating evidence suggests that histone modification is an important aspect of MLL-fusion mediated transformation and leukemogenesis (35, 36). It has been shown that wild type MLL SET domain is a methyltransferase, modifying histone H3 on lysine 4 (H3K4), and positively regulating gene expression of multiple Hox genes (37). In addition, MLL mediated transcriptional regulation involves recruitment of HAT, such as CBP (38) and MOF (39). Furthermore, DOT1L, a histone methyltransferase that methylates lysine 79 on histone H3 (H3K79), has been associated with multiple MLL-fusion partners such as AF9, AF10, AF17, and ENL (40–42), and has emerged as an attractive therapeutic target (36). Several groups have used small molecule inhibitors to demonstrate the feasibility of pharmacological inhibition of DOT1L enzymatic activity in preclinical models of MLL-rearranged leukemia (43–45) and are now under clinical investigation in a phase I study for adults with advanced hematologic malignancies, including acute leukemia with rearrangement of the MLL gene (NCT01684150). One inhibitor in particular, EPZ-5676, has shown potent activity in its ability to selectively inhibit the DOT1L histone methyltransferase, resulting in cell death of acute leukemia cell lines harboring MLL translocations as well as complete tumor regression in a rat xenograft model of MLL-rearranged leukemia following continuous iv infusion of EPZ-5676 (45).

In order to identify novel mutations in relapsed ALL, Mullighan and colleagues performed targeted resequencing of 300 genes in 23 matched relapse-diagnosis B-ALL pairs (30). The authors identified novel mutations in *CREBBP*, a gene encoding the transcriptional coactivator CREB binding protein with HAT activity. The overall frequencies of these sequence and/or deletional mutations were 18.3% in relapse cases (30). However, particularly high incidences of somatic CREBBP alterations (63%) were found in the high hyperdiploidy relapse cases. Of note, the majority of these mutations occurred in the HAT domain (27). Although less common, mutations in other important epigenetic regulators were also seen such as *NCoR1* (Nuclear corepressor complex), *EP300* (a paralog of *CREBBP)*, *EZH2* (histone methyltransferase gene), and *CTCF* (zinc finger protein involved in histone modifications) (30). Additionally, transcriptome sequencing has identified relapse-specific mutations in *CBX3* (encoding heterochromatin protein), *PRMT2* (gene encoding protein arginine methyltransferase 2), and *MIER3* (involved in chromatin binding); providing further evidence of aberrant epigenetic mechanisms that play a role at relapse (46).

Epigenetic alterations are not only restricted to B-ALL, but are a notable feature of T-ALL, particularly the aggressive subtype early T-cell precursor (ETP) ALL. Whole genome sequencing of 12 cases of ETP ALL identified mutations in genes encoding components of the polycomb repressor complex 2 (PRC2), including deletions and sequence mutations of *EZH2*, *SUZ12*, and *EED* (47). Loss of function mutations and deletions of *EZH2* and *SUZ12* genes have also been found in T-ALL, where authors implicate the tumor suppressor role of the PRC2 complex (48).

In addition to the discovery of somatic mutations in epigenetic machinery in ALL, mRNA expression of HDACs has been shown to be dysregulated. Higher mRNA expression of HDAC7 and HDAC9 in a study of 94 childhood ALL cases was shown to correlate with poor prognosis (49). Similarly, another group identified the correlation of HDAC4 overexpression with prednisone poor response, T-ALL phenotype, and a high initial WBC (50). Given the compelling evidence of HDAC’s involvement in tumor development and progression, inhibitors of HDACs have emerged as an attractive therapeutic option in hematologic malignancies (4, 51). Through a connectivity map search (52) for agents, which could potentially reverse the characteristic gene expression signature specific for relapse ALL (3, 53) and potentially endow chemosensitivity, vorinostat (HDACi) was identified as the most promising candidate (4). In fact, vorinostat not only modulated the gene expression signature characteristic of relapse in ALL cell lines and patient samples, but showed a synergistic effect when given sequentially with chemotherapy (4). The fact that vorinostat showed significant alteration of gene expression correlating with histone modifications, indicates that the perturbation of histone marks may have a key role in aberrant gene regulation at relapse. Bachmann and colleagues have reported glucocorticoid resistance associated with epigenetic silencing of the *BIM* gene in pediatric ALL and showed synergistic effect of vorinostat with dexamethasone in both *in vitro* and *in vivo* models (54). The potential importance of these changes is highlighted by the promising activity of several other drugs from the same class that target epigenetic alterations (55).

In summary, similar to the influence DNA hypermethylation has in pediatric ALL leukemogenesis, maintenance, and relapse, aberrant epigenetic changes involving histones have been associated with disease progression and relapse in ALL. With growing experience using HDACi in hematologic malignancies, including pediatric trials (NCT01483690, NCT01321346), the impact of these agents will become clearer as well as their role in future relapse and upfront ALL studies.

## MicroRNA Alterations

MicroRNAs are a class of small endogenous single stranded non-coding ribonucleic acids (RNA) composed of roughly 22 nucleotides that are primarily involved in post-transcriptional gene regulation. miRNAs play a critical regulatory role in targeting mRNAs for cleavage or translational repression, with greater than 1,000 miRNAs currently identified in the human genome (56). MicroRNA genes are preferentially localized to CpG islands, which leads to the plausible mechanism that they can be controlled through aberrant epigenetic regulation (e.g., hypermethylation, histone modification) (57).

Altered expression of miRNAs has been implicated in leukemogenesis and appears to have the ability to influence critical growth regulatory pathways in ALL (58–61). An example of the functional impact miRNA can have in B-cell ALL was reported with the restoration of miR-196b expression, which led to significant down-regulation of c-myc and its effector genes *fh*TERT, *Bcl-2*, and *AATF*, suggesting a tumor suppressor function role for miR-196b (62). Some specific miRNAs that have been implicated in pediatric ALL include miRNA (miR) miR-34, miR-128, miR-142, and miR-181, all reported to be over expressed (58, 63, 64) and miR-100 and miR-196b, both under expressed (59, 63). Schotte and colleagues investigated 397 miRNAs using qRT-PCR in 81 pediatric ALL cases in comparison to 17 normal CD34^+^ stem cell controls (65). Unique miRNA signatures were identified for various ALL sub-types including *ETV6-RUNX1*, MLL-rearranged, T-ALL, hyperdiploidy, and *E2A-PBX1*. Overall, expression of miR-143 and miR-140 were found to be 70- and 140-fold lower in the B-ALL samples compared to controls (*p*_FDR_ = 0.0007 and *p*_FDR_ = 0.001, respectively). Hyperdiploid samples showed a clustering of high expression of miR-98, miR-222, miR-223, and miR-511 and the *ETV6-RUNX1* cases had a 5- to 1700-fold increase expression in miR-99a, miR-100, miR-125b, and miR-383 compared to controls (*p*_FDR_ < 0.001). Together these findings lend support for epigenetic alterations involving miRNAs in the leukemogenesis of some of the more common variants of pediatric ALL.

Aberrant miRNA expression has been implicated in leukemia drug resistance and lower event-free survival (EFS). Schotte and colleagues identified a lower expression of miR-454 (1.9-fold lower) in leukemia blasts with l-asparaginase resistance (*p*_FDR_ = 0.017) and patient samples resistant to vincristine and daunorubicin were found to have over expression of miR-99a, miR-100, and miR-125b (14- to 25-fold) (*p*_FDR_ ≤ 0.002 and *p*_FDR_ < 0.05, respectively) (65). In terms of EFS, six miRNAs (miR-33, -215, -369-5p, -496, -518d, and -599) were associated with worse survival (HR 1.3–1.52, 95% CI 1.01–2.04; 0.003 ≤ *p* ≤ 0.046) and another eight (miR-10a, -134, -214, -484, -572, -580, -624, and -627) with greater EFS (HR 0.59–0.82, 95% CI 0.41–0.99, 0.004 ≤ *p* ≤ 0.045) (65). The authors concluded that the miRNAs associated with a more favorable outcome likely had tumor suppressor activity through their signaling of apoptosis (miR-10a), inhibition of proliferation (miR-10a and miR-214), and oncogene *SOX2* down-regulation (miR-134).

In a report of 18 matched-pair diagnosis and relapse (*n* = 8) or diagnosis and remission (*n* = 10) pediatric ALL samples, data was summarized for the most differentially expressed miRNAs (66). Down-regulation of miR-23a and miR-223 was observed at time of relapse compared to remission whereas miR130b, -181, and -708 were over expressed at relapse. Specifically, the expression of miR-708 was greater in relapse samples and lower in remission samples when compared to diagnosis whereas miR-223 was up-regulated in remission samples compared to diagnosis and confirmed with qRT-PCR. These two miRNAs at diagnosis along with miR-27a were shown to correlate significantly with 3-year relapse-free survival (*p* = 0.0483, 0.0079, and 0.0024, respectively) and thus could potentially be used as prognostic biomarkers for newly diagnosed patients. The functional impact these miRNAs had on gene expression was described as well with targets identified for *BMI1*, transcription factor necessary for hematopoietic stem cell and leukemia stem cell self-renewal, in miR-27a and miR-128b as well as E2F1, master cell cycle regulator, a target of miR-223. The variations in miRNA expression that exist between diagnostic, remission, and relapse samples identified by Han and colleagues suggest that critical epigenetic mechanisms exist through these non-coding miRNAs that may assist in driving leukemogenesis and disease recurrence.

In an analysis of 353 diagnostic bone marrow samples from patients with ALL (<15 years of age, *n* = 179), 65% had at least one of 13 previously identified miRNAs hypermethylated (67). These 13 miRNAs were found to be regulated by methylation and histone modification and associated with a closed chromatin conformation of 11 CpG islands close to where the 13 miRNAs resided. The hypermethylation was associated with miRNA under expression but could be reversed with decitabine.

In summary, aberrant miRNA expression, particularly secondary to methylation, is a common finding in ALL. These data support that epigenetic modifications of specific miRNAs are associated with chemotherapy resistance and clinical outcomes. As these modifications can be secondary to DNA hypermethylation (65, 68–71), exposure to agents such as DNMTi could reverse the aberrant expression, normalize miRNA levels, and ultimately lead to improved clinical outcomes.

## Clinical Trials Investigating Epigenetic Modifying Therapies in Pediatric ALL

The majority of clinical experience using epigenetic modifying agents in the treatment of acute leukemia has been in adults (72, 73). The Children’s Oncology Group (COG) piloted a phase I study investigating decitabine (10 mg/m^2^/day × 5 days/week × 2 weeks) in children with relapsed/refractory acute leukemia that closed prematurely due to low patient accrual (NCT00042796, unpublished). No maximum tolerated dose (MTD) was identified and 5/15 patients reported grade 3/4 cytopenias (anemia, thrombocytopenia, and leukopenia) that were possibly related to the study drug.

Similar to the DNMTi, HDACi (e.g., vorinostat, panobinostat) have been studied in the treatment of acute leukemia, primarily as single agents and almost exclusively in adults (74, 75). The COG completed a phase I study investigating vorinostat in combination with 13 cis-retinoic acid in children with refractory/recurrent solid tumors and vorinostat alone for patients with refractory leukemia (76). Six patients with refractory leukemia were enrolled with 2 DLTs reported at the solid tumor MTD (230 mg/m^2^/day) including an elevated AST (*n* = 1), hyperbilirubinemia (*n* = 1), elevated GGT (*n* = 1), and hypokalemia (*n* = 1). As the solid tumor MTD for vorinostat did not appear tolerable for patients with hematologic malignancies, there was no further dose finding attempt in this study. Currently, there is a phase I study of panobinostat in children with refractory hematologic malignancies open through the therapeutic advances in childhood leukemia and lymphoma (TACL) Consortium (NCT01321346).

The first study incorporating a DNMTi and HDACi followed by chemotherapy for children and adults with relapsed/refractory ALL was recently completed (72). In this phase II trial, decitabine (15 mg/m^2^/day) and vorinostat (230 mg/m^2^ divided BID) were given over four consecutive days prior to re-induction chemotherapy (vincristine, prednisone, PEG-asparaginase, doxorubicin) (NCT00882206) (72). Thirteen eligible patients enrolled with a median age of 16 (range, 3–54) years. There was a single toxic death occurring on study attributed to the chemotherapy regimen, which included a grade five hemorrhage/bleeding (*n* = 1). A second patient experiencing grade five hypoxia/acute respiratory distress died on day 4 of study attributed to disease progression (*n* = 1). There were an additional 14 grade 3/4 serious adverse events, which were at least possibly attributed to decitabine or vorinostat, the most common being fever with neutropenia (*n* = 2) and infection (blood) with neutropenia (*n* = 5). Results of the eight patients evaluable for response, identified a CR rate of 50% (*n* = 4/8) (95% CI 15.7–84.3%) and an overall response rate (CR + PR) of 75% (*n* = 6/8) (95% CI 34.9–96.8%). As well, minimal residual disease (MRD) negativity by flow cytometry was observed in 4/8 patients (50%, CI: 15.7–84.3%). Five of the eight patients who completed the study proceeded to allogeneic hematopoietic cell transplantation (four in second CR and one in third CR). Three patients succumbed to transplant related deaths without evidence of leukemia while the remaining two patients remain alive with no evidence of disease. Based on the results of this study, a pediatric trial for relapse/refractory ALL combining decitabine and vorinostat with re-induction chemotherapy is currently open through the TACL Consortium (NCT01483690; R21CA161688-01).

## Summary

Underlying epigenetic alterations in pediatric ALL are common events, which appear to be more common at relapse than diagnosis. Thus children with relapse ALL may be an ideal population for clinical trials incorporating epigenetic modifying agents aimed at reversing these aberrant signatures. Whether such trials will lead to improved clinical outcomes has yet to be determined but early findings in studies incorporating these agents have been encouraging.

In conclusion, leukemogenesis of pediatric ALL is heavily influenced by epigenetics, particularly DNA hypermethylation, histone modification, and alterations in miRNA expression. Epigenetic modifying agents such as DNMTi and HDACi as well as newer therapies (e.g., histone methyltransferase inhibitors) are now being incorporated into early phase clinical trials for relapse leukemia. As more trials for children with relapse ALL, incorporating epigenetic therapies into standard and/or novel salvage regimens, are developed and completed, we will have a better understanding as to which patients might benefit the most using this approach and ultimately where these agents may be best served in treating pediatric ALL.

## Conflict of Interest Statement

The authors declare that the research was conducted in the absence of any commercial or financial relationships that could be construed as a potential conflict of interest.
